# Analyses of *MMP20* Missense Mutations in Two Families with Hypomaturation Amelogenesis Imperfecta

**DOI:** 10.3389/fphys.2017.00229

**Published:** 2017-04-20

**Authors:** Youn Jung Kim, Jenny Kang, Figen Seymen, Mine Koruyucu, Koray Gencay, Teo Jeon Shin, Hong-Keun Hyun, Zang Hee Lee, Jan C.-C. Hu, James P. Simmer, Jung-Wook Kim

**Affiliations:** ^1^Department of Molecular Genetics and Dental Research Institute, School of Dentistry, Seoul National UniversitySeoul, Korea; ^2^Department of Pediatric Dentistry and Dental Research Institute, School of Dentistry, Seoul National UniversitySeoul, Korea; ^3^Faculty of Dentistry, Department of Pedodontics, Istanbul UniversityIstanbul, Turkey; ^4^Department of Cell and Developmental Biology and Dental Research Institute, School of Dentistry, Seoul National UniversitySeoul, Korea; ^5^Department of Biologic and Materials Sciences, University of Michigan School of DentistryAnn Arbor, MI, USA

**Keywords:** amelogenesis imperfecta, enamelysin, proteinase, enamel, matrix, hypomaturation

## Abstract

Amelogenesis imperfecta is a group of rare inherited disorders that affect tooth enamel formation, quantitatively and/or qualitatively. The aim of this study was to identify the genetic etiologies of two families presenting with hypomaturation amelogenesis imperfecta. DNA was isolated from peripheral blood samples obtained from participating family members. Whole exome sequencing was performed using DNA samples from the two probands. Sequencing data was aligned to the NCBI human reference genome (NCBI build 37.2, hg19) and sequence variations were annotated with the dbSNP build 138. Mutations in *MMP20* were identified in both probands. A homozygous missense mutation (c.678T>A; p.His226Gln) was identified in the consanguineous Family 1. Compound heterozygous *MMP20* mutations (c.540T>A, p.Tyr180^*^ and c.389C>T, p.Thr130Ile) were identified in the non-consanguineous Family 2. Affected persons in Family 1 showed hypomaturation AI with dark brown discoloration, which is similar to the clinical phenotype in a previous report with the same mutation. However, the dentition of the Family 2 proband exhibited slight yellowish discoloration with reduced transparency. Functional analysis showed that the p.Thr130Ile mutant protein had reduced activity of MMP20, while there was no functional MMP20 in the Family 1 proband. These results expand the mutational spectrum of the *MMP20* and broaden our understanding of genotype-phenotype correlations in amelogenesis imperfecta.

## Introduction

Non-syndromic amelogenesis imperfecta (AI), hereditary enamel defects, can be divided into 3 major categories based on the quantity and quality of the enamel (Witkop, 1988). In hypoplastic AI, the enamel is thin with interdental spacing and the affected individuals are often sensitive to thermal changes and possess an increased tendency of anterior open bite (Ravassipour et al., 2005). In hypocalcification AI, the affected enamel is extremely soft with normal thickness prior to tooth eruption, which may be lost rapidly after eruption leaving the remaining enamel rough, discolored, and thin. Hypomaturation AI is caused by failures during the maturation stage of amelogenesis. The resulting phenotype is characteristically (dark) brown or yellowish discolored less mineralized enamel with normal thickness. But because the enamel is not matured well, prolonged attrition can result in excessive enamel wear facets or localized enamel fractures (Wright et al., 2011). However, definitive characterization of the phenotype may be challenging in some cases. Therefore, a broader classification scheme with two categories has been used: hypoplastic AI and hypomineralized AI. The hypomineralized AI includes hypocalcification AI and hypomaturation AI (Prasad et al., 2016).

To date, mutations in more than 17 genes are involved in non-syndromic AI. Hypoplastic AI can be caused by mutations in *AMELX* (MIM: 300391), *ENAM* (MIM: 606585), *AMBN* (MIM: 601259), *LAMB3* (MIM: 150310), *LAMA3* (MIM: 600805), *COL17A1* (MIM: 113811), *ITGB6* (MIM: 147558), and *ACPT* (MIM: 606362) (Lagerstrom et al., 1991; McGrath et al., 1996; Rajpar et al., 2001; Yuen et al., 2012; Kim et al., 2013; Wang et al., 2014; Poulter et al., 2014a,b,c; Seymen et al., 2016). Autosomal dominant hypocalcification AI is caused by mutations in *FAM83H* (MIM: 611927) (Kim et al., 2008). Some *AMELX* mutations can cause hypomaturation AI with enamel hypoplasia (Hart et al., 2000). Recessive mutations in *SLC24A4* (MIM: 609840), *WDR72* (MIM: 613214), *MMP20* (MIM: 604629), *KLK4* (MIM: 603767), and *GPR68* (MIN: 601404) cause hypomaturation AI (Wright et al., 2003; Hart et al., 2004; Kim et al., 2005; El-Sayed et al., 2009; Parry et al., 2013, 2016). Clinical phenotype caused by autosomal recessive mutations of *C4orf26* (MIM: 614829) and autosomal dominant mutation of *AMTN* (MIM: 610912) were reported as hypomineralization AI (Parry et al., 2012; Smith et al., 2016).

Two proteinases secreted by ameloblasts during mammalian enamel formation are matrix metalloproteinase 20 (MMP20, enamelysin) and kallikrein 4 (KLK4) (Hu et al., 2002). MMP20 is the early protease expressed by ameloblasts throughout the secretory stage and early maturation stage of amelogenesis. KLK4 is the late protease expressed by ameloblasts from the transition stage to the maturation stage. Lack of proteinase function in the maturing enamel matrix prevents proper degradation and removal of the enamel matrix proteins resulting in enamel hypomaturation AI.

Here we report the identification of *MMP20* mutations in two Turkish families with hypomaturation AI by whole exome sequencing and the mutational effect on the protein secretion and proteolytic activity.

## Materials and methods

### Identification and enrollment of AI families

Clinical and radiographic examinations of the probands and their available family members were performed, and blood samples were collected with the understanding and written consent of each participant according to the Declaration of Helsinki. Affected individuals were healthy, except hypomaturation enamel defects. The study protocol was independently reviewed and approved by the Institution Review Board at the Seoul National University Dental Hospital, the University of Istanbul and the University of Michigan.

### DNA isolation and whole-exome sequencing

Genomic DNA was isolated from peripheral whole blood. The purity and concentration of the DNA were quantified by spectrophotometry measurement and the OD_260_/OD_280_ ratio obtained. Whole-exome sequencing was performed with the DNA sample of the probands using Illumina HiSeq 2000 platform. The NimbleGen (Roche Diagnostics, Indianapolis, IL, USA) exome capture reagent was used for exome capturing.

### Autozygosity mapping

The affected individuals in family 1 (IV:3 and IV:4) were genotyped with the Affymetrix Genome-Wide Human SNP array 6.0 (DNALINK INC., Seoul, Korea). The annotated SNP files were analyzed with HomozygosityMapper (http://www.homozygositymapper.org/) (Seelow et al., 2009) to identify the shared regions of homozygosity in the affected individuals.

### Segregation analysis by polymerase chain reaction (PCR)

The sequence variations in the *MMP20* gene and segregation within each family was confirmed by Sanger sequencing with primers and conditions described previously (Kim et al., 2005). PCR amplifications were done with the HiPi DNA polymerase premix (Elpis Biotech, Daejeon, Korea), and DNA sequencing was performed at a DNA sequencing center (Macrogen, Seoul, Korea).

### Cloning and mutagenesis of the *MMP20* cDNA

Human *MMP20* cDNA, previously cloned into the pcDNA3.1 vector, was used to introduce the identified mutations using PCR mutagenesis (sense: 5′-TACCGTTGCTGCTCAAGAATTTGGCCATGC, antisense: 5′-GCATGGCCAAATTCTTGAGCAGCAACGGTA for the p.His226Gln and sense: 5′-GAATATCTAAATACATACCTTCCATGAGTT, antisense: 5′-AACTCATGGAAGGTATGTATTTAGATATTC for the p.Thr130Ile) (Lee et al., 2010). Sequences of normal and mutant *MMP20* pcDNA3.1 vectors were confirmed by direct plasmid sequencing.

### Transfection

HEK293T cells were grown and maintained in DMEM supplemented with 10% FBS and antibiotics in a 5% CO_2_ atmosphere at 37°C. Cells at ~2 × 10^5^ quantity were seeded in each well of the 6-well culture dish. Each plasmid construct at 2 ug quantity was transiently transfected into HEK293T cells with Genjet *in vitro* DNA transfection reagent (SigmaGen Laboratories, Ijamsville, MD, USA). The culture medium of each well was harvested after 30 h of incubation and concentrated using Amicon ultra-4 centrifugal filter units (Millipore, Bedford, MA, USA).

### Zymography

Four ml of conditioned medium from the culture was collected and concentrated to 50 ul. The concentrated media of 20 ul was mixed with 4 ul of 5x non-reducing buffer, then loaded onto the 11% SDS-polyacrylamide gel with β-casein (Sigma-Aldrich, St. Louis, MO, USA) as a substrate. The zymogram was developed, stained with Coomassie brilliant blue R-250 staining solution (Bio-rad, Hercules, CA, USA) for 20 min, and visualized after incubation in a destaining solution (10% MeOH, 10% acetic acid) for 3 h.

### Western blot

Concentrated media and cell lysates were run on the 11% SDS-polyacrylamide gel and subjected to Western blotting. Specifically, 50 ug cell lysate from each sample and 10 ul of concentrated media were used. After gel transfer to the PVDF membrane and blocking, MMP20 was detected by incubating the membrane with primary antibody overnight at 4°C and with secondary antibody for 2 h at room temperature. The primary antibodies used were a rabbit polyclonal anti-MMP20 antibody (ab39038, abcam plc., Cambridge, UK) and a mouse monoclonal anti-ACTB antibody (A2228, Sigma-Aldrich, St. Louis, MO, USA); both of which were diluted in 1:10,000.

## Results

### Clinical phenotype

**Figure 1:**
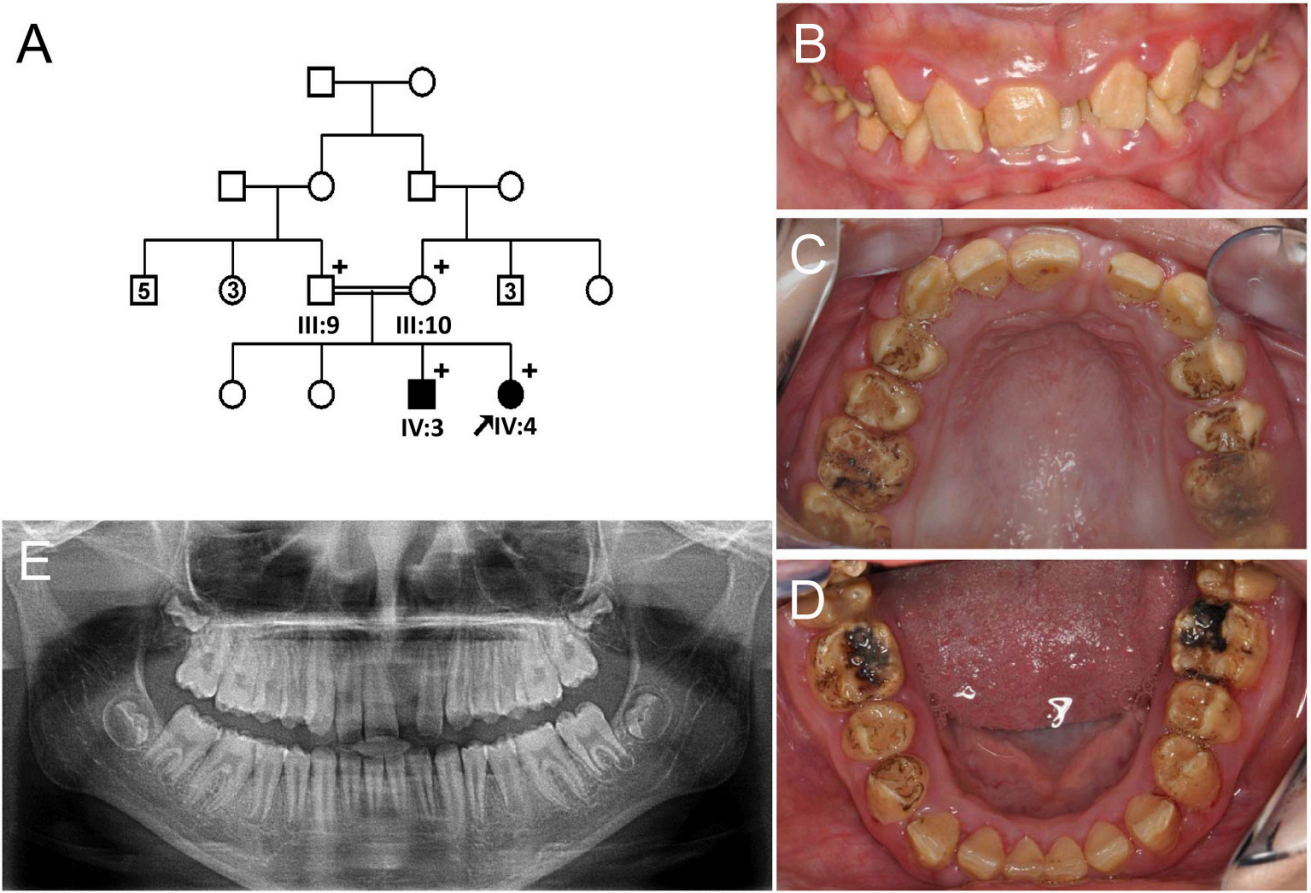
**Pedigree and clinical phenotype of the proband of Family 1. (A)** Pedigree of family 1. **(B)** Frontal view of the proband (IV:4) at age 11. **(C)** Maxillary occlusal view. **(D)** Mandibular occlusal view. **(E)** Panoramic radiograph of the proband at age 11. Numbers in the subject symbol indicate the number of siblings. Plus symbols indicate individuals who participated in this study.

The proband of Family 1 (IV:4) was an 11-year-old girl from a consanguineous marriage of first cousins (Figure 1A). Her prenatal and perinatal history was uneventful and her parents reported no other medical problem. Her teeth exhibited generalized brown discoloration with exogenous black pigmentation mainly on occlusal surface of the posterior teeth (Figures 1B–D). Maxillary left central incisor was lost due to trauma. The radiopacity of enamel did not contrast well with dentin in the panoramic radiograph, consistent with hypomineralization (Figure 1E). Her 24-year-old brother (IV:3) was also affected and almost all of his teeth have been reconstructed with full-coverage prosthodontics. His remaining natural teeth exhibited dark brown discoloration with exogenous pigmentation (Figure S1).

**Figure 2:**
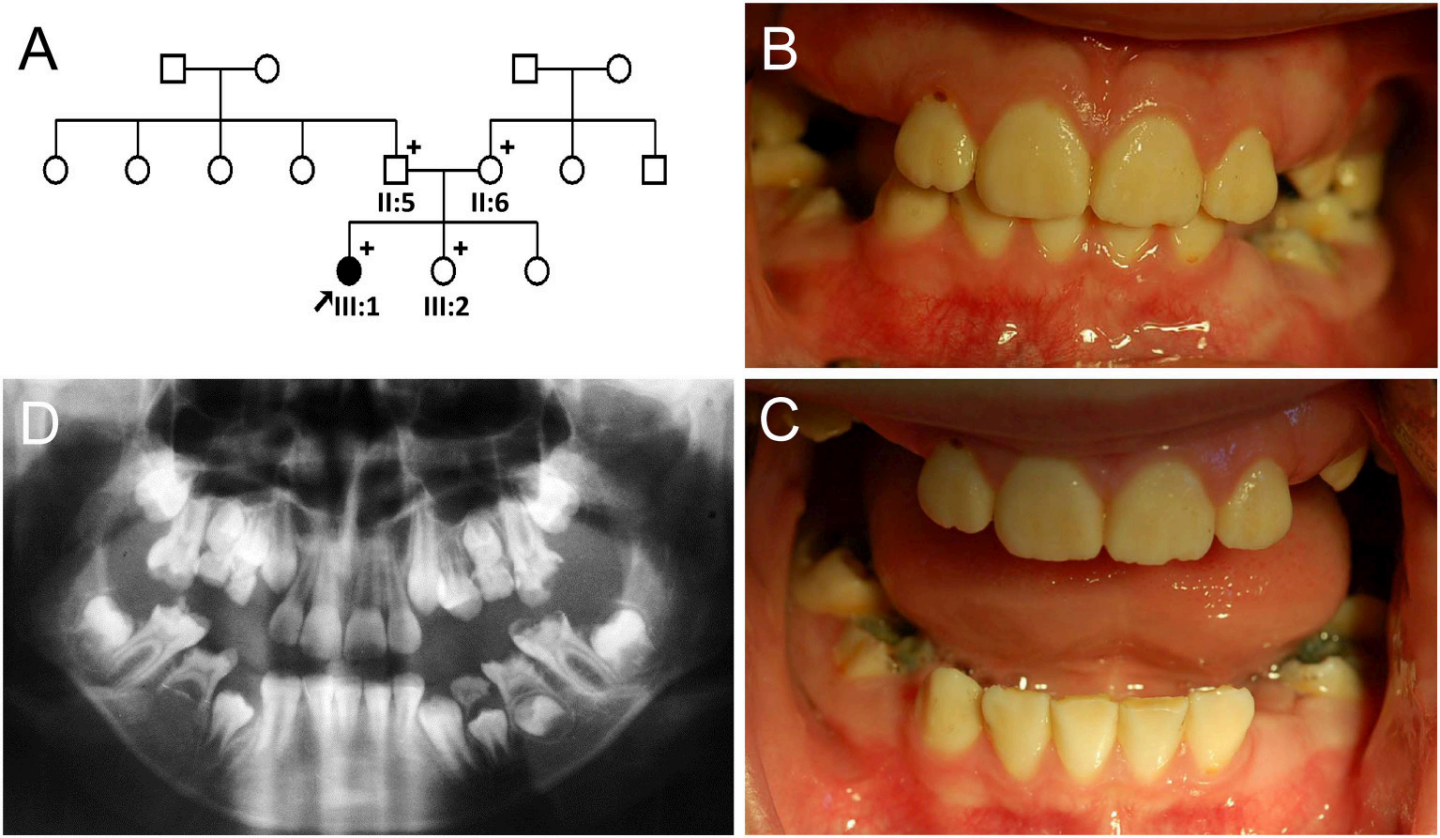
**Pedigree and clinical phenotype of the proband of Family 2. (A)** Pedigree of family 2. **(B)** Frontal view of the proband (III:1) at age 10. **(C)** Frontal view with bite open. **(D)** Panoramic radiograph of the proband at age 10. Plus symbols indicate individuals who participated in this study.

The proband of the family 2 (III:1) was a 10-year-old girl from a non-consanguineous family (Figure 2A). Her past medical history was unremarkable. Her anterior permanent teeth were not severely discolored, but slightly yellow and less transparent than normal teeth (Figures 2B,C). Her right second premolar was congenitally absent based on the panoramic radiograph (Figure 2D).

### Mutational analysis

Sequencing reads were aligned to the UCSC human reference genome (hg19) with Burrow-Wheeler Aligner, and the sequence variations were annotated by referencing dbSNP build 138, which preceded variant calling with SAMtools and GATK (Table S1). Annotated variants were filtered with the criteria of minor allele frequency of 0.01.

Autozygosity mapping of the family 1 revealed 3 shared regions of loss of heterozygosity: chr4:65,904,881–82,427,846, chr11:83,358,629–113,318,007, and chr21:11,039,570–17,728,224 (Figure S2). The exome data in the shared regions of the proband in family 1 revealed a homozygous variant in exon 5 of the *MMP20* gene (NM_004771.3: c.678T>A). This transversion of thymine to adenine changed histidine to glutamine at amino acid position 226 (p.His226Gln). There was no other variation in the known AI-causing genes and the *MMP20* mutation (c.678T>A, p.His226Gln) was previously reported as an AI-causing mutation (Ozdemir et al., 2005; Wright et al., 2011).

Whole exome sequencing of the Family 2 proband revealed compound heterozygous *MMP20* mutations (c.389C>T and c.540T>A). There was no other variation in the known AI-causing genes. A cytosine to thymine transition in exon 3 changed threonine to isoleucine at amino acid position 130 (p.Thr130Ile). This variation was listed in the Exome Aggregation Consortium (ExAC) database (rs61730849) with an allele frequency of 0.00165 (200/121176). But the frequency was relatively high (0.0294) in a certain subset of small population (ss86247256, AGI_ASP_population; Coriell Apparently Healthy Collection). In addition, it was previously reported as a disease-causing mutation (Gasse et al., 2013). The other variation, a transversion of thymine to adenine in exon 4, would introduce a premature stop codon (p.Tyr180^*^) and the mutant transcript would be degraded by the nonsense-mediated decay system. This variant was not listed in any database.

**Figure 3:**
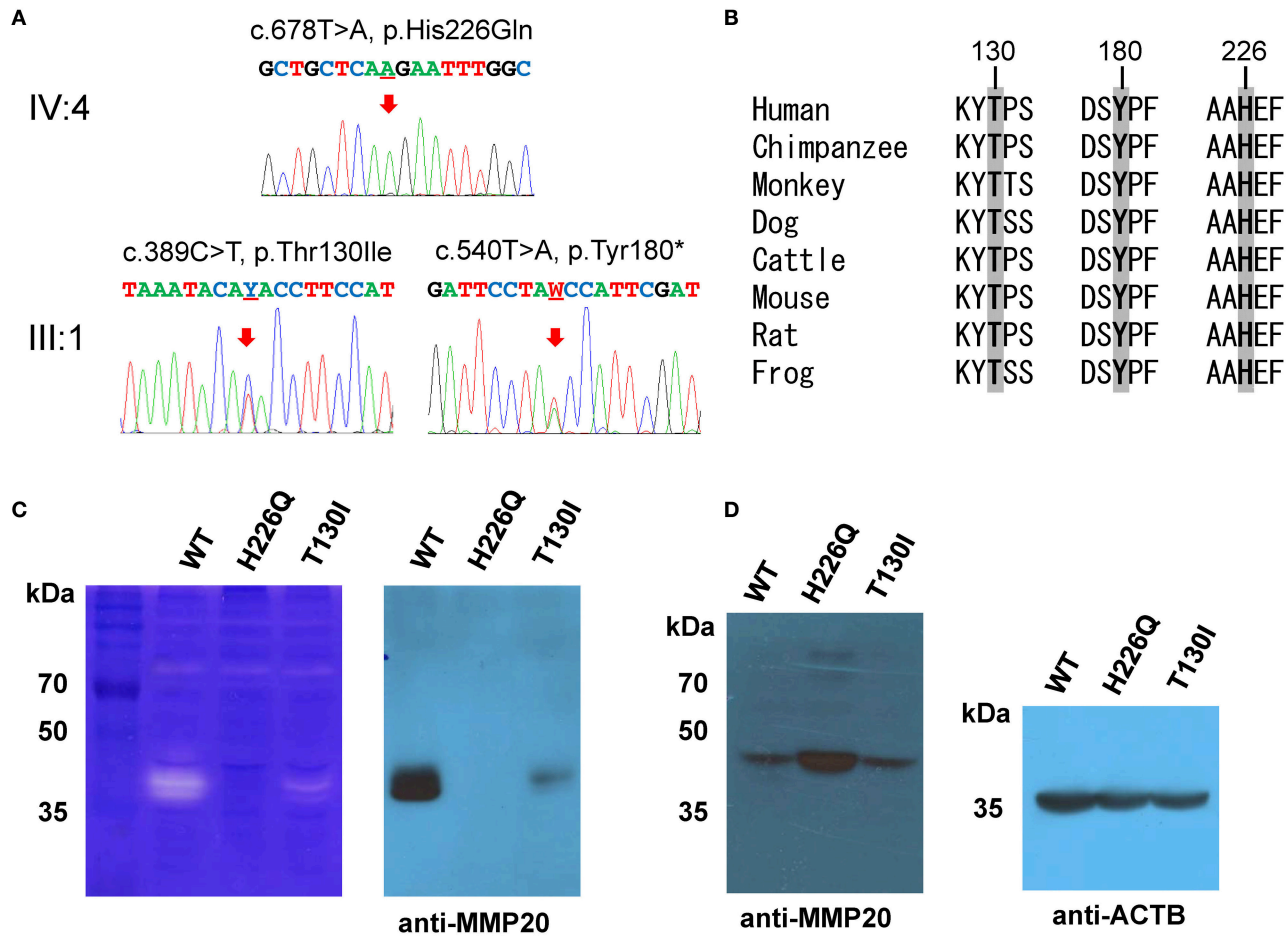
**Orthologs alignment, sequencing chromatograms and ***in vitro*** translation. (A)** Sanger sequencing chromatograms of the probands (IV:4 of family 1 and III:1 of family 2). The mutated nucleotide is indicated by a red arrow and underlined (Y; C or T and W; A or T). **(B)** Sequence alignment of vertebrate orthologs. Amino acids affected by the mutations are indicated with bold character and gray highlight. Numbers above the amino acids are based on the human MMP20 sequence. **(C)** Casein zymography indicated that the p.Thr130Ile mutant protein retains proteolytic function, but the p.His226Gln mutant protein has no proteolytic activity. Western blot of the conditioned media revealed that the secretion of the p.Thr130Ile mutant protein into the culture media was greatly reduced, but the p.His226Gln mutant protein cannot be secreted at all. **(D)** Western blot of the cell lysate demonstrated that the p.His226Gln mutant protein remained in the cell. (ACTB: beta actin).

Segregation within the families by Sanger sequencing confirmed that the nonsense mutation (c.540T>A, p.Tyr180^*^) was transmitted paternally and the missense mutation (c.389C>T, p.Thr130Ile) was transmitted maternally to the proband (Figure 3A, Figure S3). These amino acids at the mutation sites (Thr130, Tyr180, and His226) are strictly conserved among eutherian mammal orthologs (Figure 3B).

Western blotting and zymography determining the function of the MMP20 mutants demonstrated that the p.Thr130Ile mutant protein was secreted at a reduced amount and had proteolytic activity. Western blot of cell lysate revealed that the p.His226Gln mutant protein was retained in the cell and likely not able to be secreted (Figures 3C,D).

## Discussion

MMP20 is one of 23 human matrix metalloproteinases. It processes structural enamel matrix proteins into functional fragments in the secretory stage and facilitates the removal of those proteins during the maturation stage. *MMP20* gene is located in a cluster with 7 other MMPs on chromosomal location 11q22.3. *MMP20* encodes a 483-amino-acid protein, which has a signal peptide (Met1 to Ala22), a prodomain (Ala23 to Asn107), a catalytic domain (Tyr108 to Gly271), a linker (Pro272 to Leu295) and a hemopexin domain (Cys296 to Cys483) (Llano et al., 1997).

The homozygous missense mutation (c.678T>A, p.His226Gln) identified in Family 1 was previously reported (Ozdemir et al., 2005; Wright et al., 2011). His226 is one of the three histidine residues involved in the coordination of zinc ion at the active site (Llano et al., 1997). This study showed that the p.His226Gln mutant protein cannot be secreted into the developing extracellular matrix, probably due to a structural change in the core area of the protein.

The Family 2 proband had a paternal nonsense mutation (c.540T>A, p.Tyr180^*^) and a maternal missense mutation (c.389C>T, p.Thr130Ile) (Gasse et al., 2013). This novel nonsense mutation would introduce a premature stop codon in exon 4, so the mutant mRNA transcript would be degraded by the nonsense-mediated decay system. This study showed that the p.Thr130Ile mutant protein could be secreted into the developing enamel matrix and retained proteolytic function.

The functional analysis suggested that the Family 2 proband would have reduced MMP20 activity, while there's no functional MMP20 in the Family 1 proband. This reduced functional activity of MMP20 potentially explains the difference in clinical phenotype between the probands of these two families. Among nine mutations in *MMP20* gene reported to date (Kim et al., 2005; Ozdemir et al., 2005; Papagerakis et al., 2008; Lee et al., 2010; Wright et al., 2011; Gasse et al., 2013; Wang et al., 2013; Seymen et al., 2015), mutations presumed to have retained functional activity would likely present less severe discoloration compared to nullifying mutations (Table 1). The degree of discoloration could be an indicator of the enamel porosity and reflects an altered level of maturation. Therefore, such clinical feature reflecting enamel quality should be considered by clinicians when devising a treatment plan for the patient.

**Table 1:** **Disease-causing mutations of the MMP20 gene**.

**Location**	**cDNA**	**Protein**	**Mode of inheritance**	**References**
Exon 1	c.102G>A	p.Trp34^*^	AR homo	Papagerakis et al., 2008; Chan et al., 2011
Exon 2	c.359delA	p.Asn120Ilefs^*^3	AR paternal	Gasse et al., 2013
Exon 3	c.389C>T	p.Thr130Ile	AR maternal / AR homo	Gasse et al., 2013
Exon 4	c.540T>A	p.Tyr180^*^	AR paternal	This report
Exon 4	c.611A>G	p.His204Arg	AR homo	Wang et al., 2013
Exon 5	c.678T>A	p.His226Gln	AR homo	Ozdemir et al., 2005; Wright et al., 2011
Exon 6	c.910G>A	p.Ala304Thr	AR homo	Lee et al., 2010
Intron 6	c.954-2A>T	p.0 or p.Ile319^*^ or p.Ile319Serfs^*^20	AR homo	Kim et al., 2005; Wright et al., 2011
Exon 7	c.1054G>A	p.Glu352Lys	AR homo	Seymen et al., 2015

* *Sequences based on the reference sequence for mRNA (NM_004771.3) and protein (NP_004762.2), where the A of the ATG translation initiation codon is designated as nucleotide 1*.

As mutations of the *MMP20* gene are characterized, their functional impact investigated, and clinical features of the affected individuals documented, it will enhance our ability to establish genotype and phenotype correlation and provide the needed evidence to improve clinical diagnosis and management of patients with AI.

## Author contributions

Study design: FS, JH, JS, and JWK. Data collection: MK, KG, TS, HH, and ZL. Data analysis: YK, MK, KG, TS, HH, and JWK. Drafting manuscript: YK, JH, JS, and JWK. Revising manuscript content: JK, JH, JS, and JWK. Approving final version of manuscript: YK, JK, FS, MK, KG, TS, HH, ZL, JH, JS, and JWK. JWK takes responsibility for the integrity of the data analysis.

## Funding

This work was supported by grants from the National Research Foundation of Korea (NRF) grant funded by the Korea government (2014R1A2A1A11049931) and the National Institute for Dental and Craniofacial Research (DE015846).

### Conflict of interest statement

The authors declare that the research was conducted in the absence of any commercial or financial relationships that could be construed as a potential conflict of interest.
