# Supplementary material for: Analyses of MMP20 Missense Mutations in Two Families with Hypomaturation Amelogenesis Imperfecta
Source: Front Physiol. 2017 Apr 20;8:229. doi: 10.3389/fphys.2017.00229 (PMC5397402; doi:10.3389/fphys.2017.00229)
Supplement: Supplementary file 1 [file DataSheet1.DOCX]

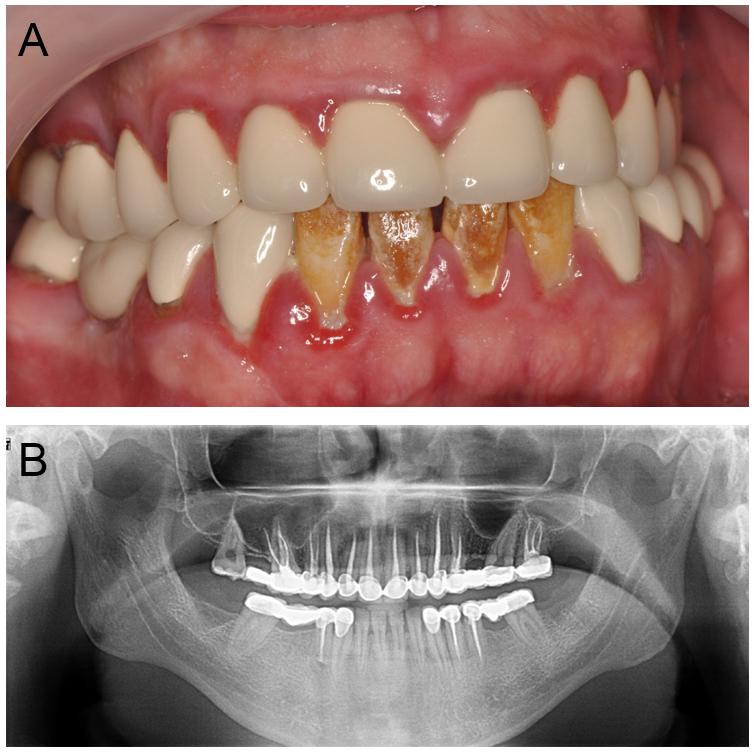


**Figure S1** Clinical photo and panoramic radiograph of affected sibling (IV:3) in family 1. (**A** and **B**) All of his teeth, except mandibular incisors, have been reconstructed with full-coverage prosthodontics.

**
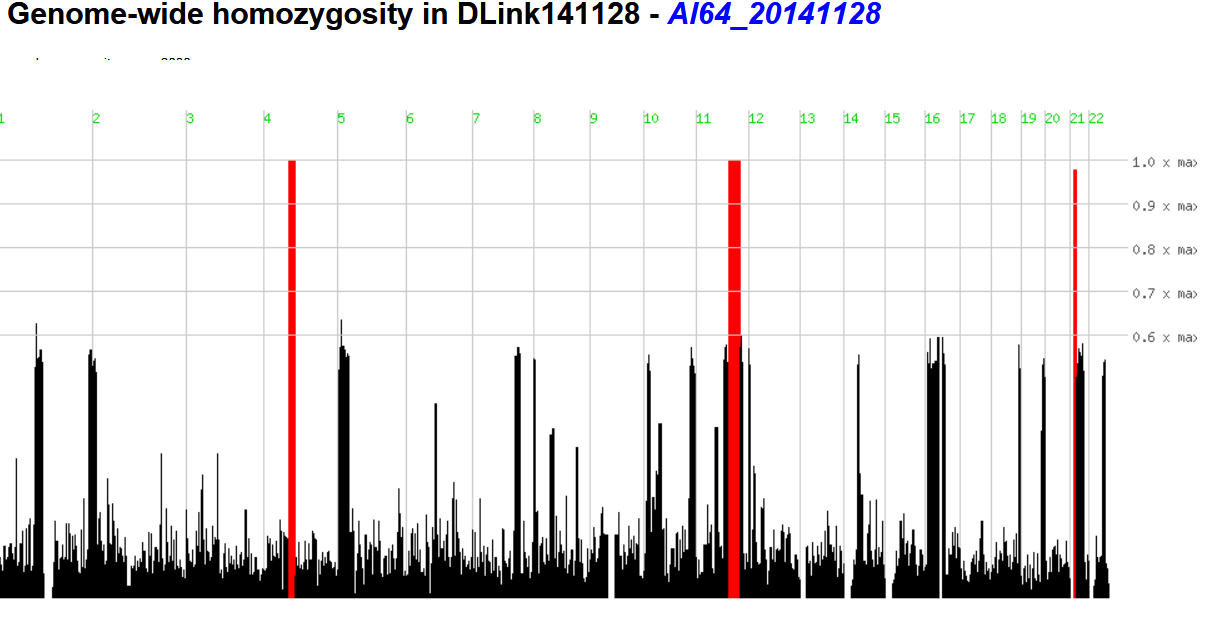
**

**Figure S2** Autozygosity mapping result with affected individuals in family 1 (IV:3 and IV:4). Autozygosity mapping result shows 3 shared regions on chromosome 4, 11, and 21 (red color). The green numbers on the top of the image indicate chromosome numbers.


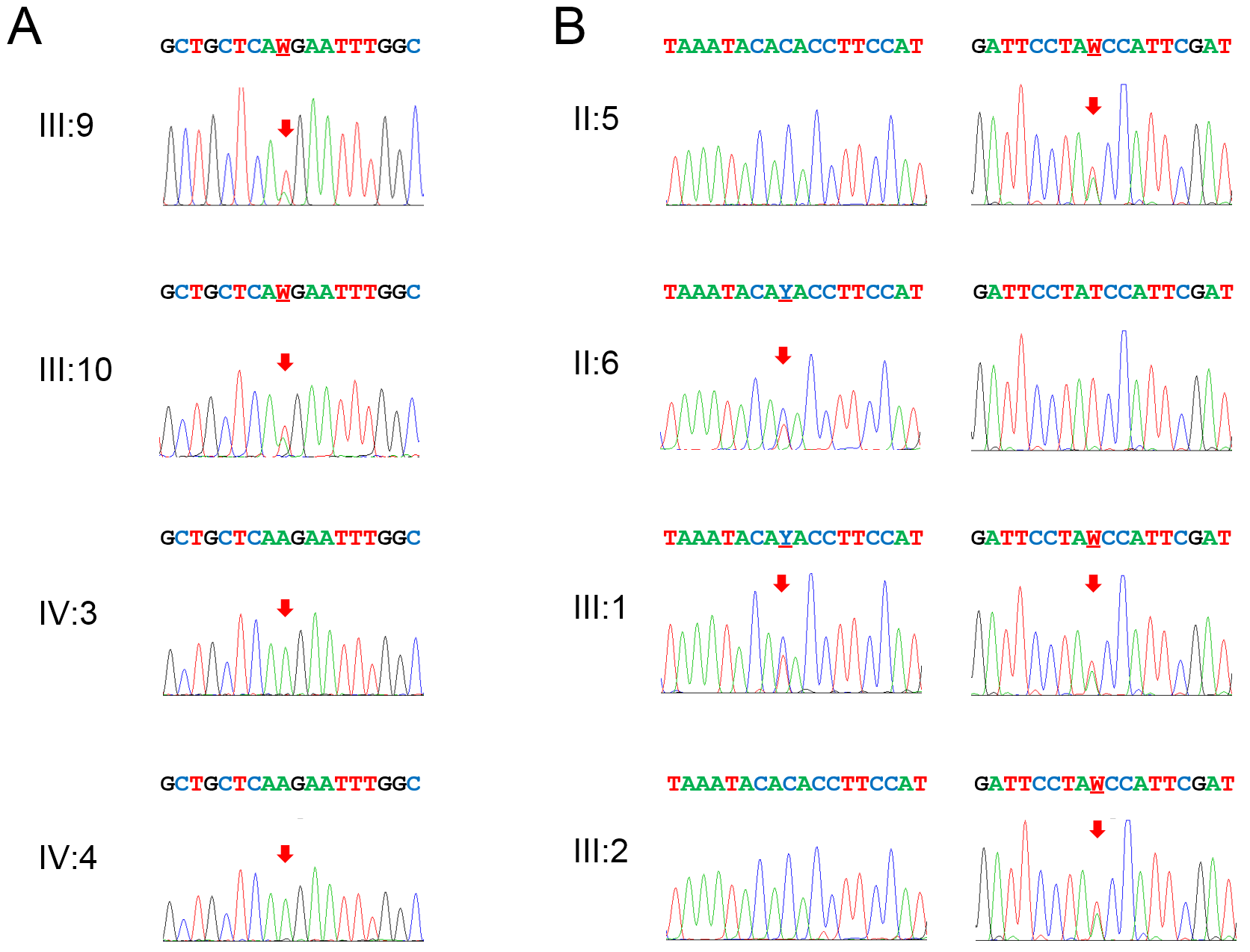


**Figure S3** Sanger sequencing chromatograms in family 1 (**A**) and family 2 (**B**). The mutated nucleotide is indicated by an underline and red arrow (Y; C or T and W; A or T).

**Table S1.** Statistics for exome sequencing.

| Exome Capture Statistics | Family 1 IV:4 | Family 2 III:1 |
| --- | --- | --- |
| Total Reads | 67750043 | 55576936 |
| Mapped Reads | 67604577 | 55489033 |
| Mapping Rate (%) | 99.79 | 99.84 |
| Read length (bp) | 74 | 74 |
| Average sequencing depth on target | 52.10 | 42.24 |
| Fraction of target covered with at least 1X (%) | 90.75 | 90.58 |
| Fraction of target covered with at least 2X (%) | 88.97 | 88.82 |
| Fraction of target covered with at least 10X (%) | 83.35 | 81.68 |
| Fraction of target covered with at least 20X (%) | 74.56 | 69.72 |
| Fraction of target covered with at least 30X (%) | 64.06 | 56.41 |
| Fraction of target covered with at least 40X (%) | 53.47 | 44.19 |
| Fraction of target covered with at least 50X (%) | 43.79 | 33.93 |
